# Disentangling the inverse relationship between cancer and Alzheimer’s or Parkinson’s disease: A systematic review on Mendelian randomization studies

**DOI:** 10.1016/j.nbd.2025.107190

**Published:** 2025-11-13

**Authors:** Khine Zin Aung, Su Su Zin, Xian Wu, Zin War Myint, Shama Karanth, Steven Estus, Christopher M. Norris, Peter T. Nelson, David W. Fardo, Erin L. Abner, Yuriko Katsumata

**Affiliations:** a Department of Biostatistics, University of Kentucky, Lexington, KY 40536, USA; b Sanders-Brown Center on Aging, University of Kentucky, Lexington, KY 40536, USA; c Department of Health Management and Policy, University of Kentucky, Lexington, KY 40536, USA; d Division of Medical Oncology, Department of Internal Medicine, Markey Cancer Center, University of Kentucky, Lexington, KY 40536, USA; e Department of Surgery, College of Medicine, University of Florida, 1600 SW Archer Rd, Gainesville, FL 32608, USA; f UF Health Cancer Center, University of Florida, 2033 Mowry Rd, Gainesville, FL 32610, USA; g Department of Physiology, University of Kentucky, Lexington, KY 40536, USA; h Department of Pharmacology & Nutritional Sciences, University of Kentucky, Lexington, KY 40536, USA; i Department of Pathology, University of Kentucky, Lexington, KY 40536, USA; j Department of Epidemiology and Environmental Health, University of Kentucky, Lexington, KY 40536, USA

**Keywords:** Alzheimer’s disease, Parkinson’s disease, Cancer, Mendelian randomization, Cognitive decline

## Abstract

**Introduction:**

Although studies have reported an inverse relationship between cancer and neurodegenerative diseases such as Alzheimer’s disease (AD) and Parkinson’s disease (PD), findings remain inconsistent. Observational studies are limited by survival bias and reverse causation. To better understand the relationship, we conducted a systematic review of Mendelian randomization (MR) studies examining both directions—assessing cancer as a risk factor for AD or PD, as well as AD or PD as exposures influencing cancer risk.

**Methods:**

We systematically reviewed MR studies investigating the causal relation between cancer and either AD or PD. Cancer could be specified as either an exposure or an outcome of interest. Articles published until August 2024 were identified, screened, and abstracted by two reviewers following the “Preferred Reporting Items for Systematic Reviews and Meta-Analyses (PRISMA)” guidelines.

**Results:**

Twelve studies met the inclusion criteria, comprising data from approximately 10,000 individuals and examining over 20 cancer types in relation to AD and PD risk. Of these, seven studies focused on AD, three on PD, and two examined both. Among nine studies on AD, an inverse association between several cancers and AD was reported, especially with breast cancer (overall and estrogen receptor-positive), with reduced odds (OR < 1) using inverse variance weighting. Studies on PD yielded inconclusive evidence of any causal relationship with cancer.

**Conclusion:**

These results highlight inverse associations between AD and breast cancers, potentially implicating hormonal signaling pathways. Despite variations in methods and GWAS datasets, consistent protective trends were observed. However, further research is required to confirm causality.

## 1. Introduction

Both Alzheimer’s disease (AD) and cancer are common clinical syndromes in older adults with high morbidity and mortality. These syndromes share several risk factors, including advancing age, lower education, ancestry, inflammation and other immune system dysregulation (Zabłocka et al., 2021; Shade et al., 2024). Given these shared risk factors, AD and cancer would intuitively be positively associated with each other; however, numerous studies have reported a negative association, indicating lower incidences of AD and other neurodegenerative diseases among people with a cancer history relative to cancer-free controls (Karanth et al., 2022; Tirumalasetti et al., 1991; Driver et al., 2012; Mezencev and Chernoff, 2020). These observations raise questions about whether the inverse association is spurious or causal and, if it is causal, what underlying biological and environmental factors may explain it.

Genetic and transcriptomic studies investigated overlapping biological pathways in both cancer and AD (Holohan et al., 2012). For example, microRNAs, including miR-9, miR-29, and miR-132, are important modulators of transcription, proliferation, apoptosis, inflammation, DNA methylation, and tau pathology that are involved in AD (and other dementias) and cancer (Holohan et al., 2012; Walgrave et al., 2021). Moreover, p53, a tumor suppressor, undergoes aggregation, mislocalization, and interaction with tau oligomers in AD brains (Farmer et al., 2025). The AD proteinopathic tau protein is normally involved in the stabilization of microtubules, and mutated tau (i.e., resulting from a mutation in the *MAPT* gene) is reported to influence the spread of cancer cells and cell growth (Wang and Mandelkow, 2016; Jimenez-Blasco et al., 2024; Rossi et al., 2018).

Although observational studies can provide valuable insights into associations, they are subject to various biases that limit the validity of causal inferences. Survival bias is a particular concern, as cancer patients may not live long enough to be at risk of developing neurodegenerative diseases such as AD or Parkinson’s disease (PD), or vice versa (Zheng et al., 2024; Sun et al., 2020). Information bias is also problematic, as patients with dementia may not be accurately monitored for cancer or may have difficulty recalling their cancer history. Since the initial report by Tirumalasetti et al. in 1991 (Tirumalasetti et al., 1991), numerous studies have attempted to address these potential biases through improved study designs and statistical methods (Driver et al., 2012). Mendelian randomization (MR) is an analytic approach used to estimate the causal relationship between an exposure and an outcome when all factors (known and unknown) that may influence both cancer and neurodegenerative disease risk cannot be accounted for (Burgess and Thompson, 2017; Burgess et al., 2020).

The gold standard for inferring causality in epidemiological studies is a randomized controlled trial (RCT) (Evans, 1998). In an RCT, participants are randomly assigned to treatment groups, and this randomization ensures that (on average) the groups are exchangeable (more formally, the probability of treatment provides no information about the probability of future outcomes) (Hernan and Robins, 2023). However, conducting an RCT to examine associations between diseases such as AD and cancer is not feasible due to ethical and practical limitations. Instead, the MR approach, which exploits the random allocation of alleles from each parent to their offspring, is used to emulate randomization assignments in an RCT. Based on Mendel’s laws of inheritance, MR treats genetic variants as instrumental variables for exposures, effectively randomizing individuals by genotype, thereby leveraging the concept of the random allocation of alleles at conception to minimize confounding bias and reverse causation bias (Davey Smith and Hemani, 2014). Recent improvements in imputation reference panels for genome-wide association studies (GWAS) have enabled MR to infer causal relationships with complex and polygenic diseases. Large-scale GWAS have identified many genetic variants linked to various diseases, facilitating MR studies that estimate causality when RCTs are not feasible.

MR plays an important role in observational studies, helping to elucidate causal relationships between AD and cancer, and identifying underlying therapeutic targets and candidates for drug repositioning for both diseases (Zhu et al., 2024; Pan and Jing, 2025; Wu et al., 2021). Two-sample MR, in particular, utilizes summary statistics from large-scale GWAS for each trait, leveraging genetic variants as instrumental variables, even for small effect sizes. In this review, we summarize recent two-sample MR (hereafter referred to as MR) studies to explore the causal relationships and potential biological connections between AD/PD and cancer.

## 2. Methods

### 2.1. Literature search

A systematic literature search was conducted using PubMed, Embase, and Web of Science, with an open time frame (articles published up to 1 August 2024). To address variations in spelling and synonyms, various query terms related to “Alzheimer’s disease”, “Parkinson’s Disease”, “cancer”, and “Mendelian randomization” were utilized, adhering to the Preferred Reporting Items for Systematic Reviews and Meta-Analyses (PRISMA) framework. The search strategy is provided in Supplementary Table 1. Unpublished articles, articles written in non-English languages, irrelevant research topics, studies not using MR, and conference abstracts were excluded.

A total of 315 articles were identified through the literature search: 114 from PubMed, 96 from Embase, and 105 from Web of Science. All the articles were imported into Covidence (www.covidence.org) and EndNote (version 20.0.0.17096) for screening. After removing duplicates, 207 articles remained. Title and abstract screening was conducted by one reviewer (K.Z.A.), resulting in 75 articles selected for full-text review. The remaining 132 were excluded as they did not align with the primary research question. Full-text screening and final study selection were then performed independently by two reviewers (K.Z.A. and S.S.Z.). Any disagreements were resolved through consultation with a third reviewer (Y.K.).

### 2.2. MR statistics extraction

We summarized MR studies investigating the relationship of various types of cancer with AD and PD in Tables 1 and 2, respectively. Both tables include the surname of the first author, the year of publication, and the exposure and outcome in the respective MR analyses. Subsequently, we reported odds ratios (ORs) with 95 % confidence intervals (CIs) estimated using the inverse-variance weighted (IVW) method as the primary findings, including the exposure and outcome, as well as the number of genetic variants used in the analyses. The IVW method is the most reliable and has the greatest statistical power when the three instrumental assumptions (relevance, independence, and exclusion restriction) of MR are satisfied (Burgess et al., 2013). MR statistics were extracted independently by two reviewers (K.Z.A. and S.S.Z.). The final check was conducted by a third investigator (Y.K.).

## 3. Results

### 3.1. Study summary

**Fig. 1.**
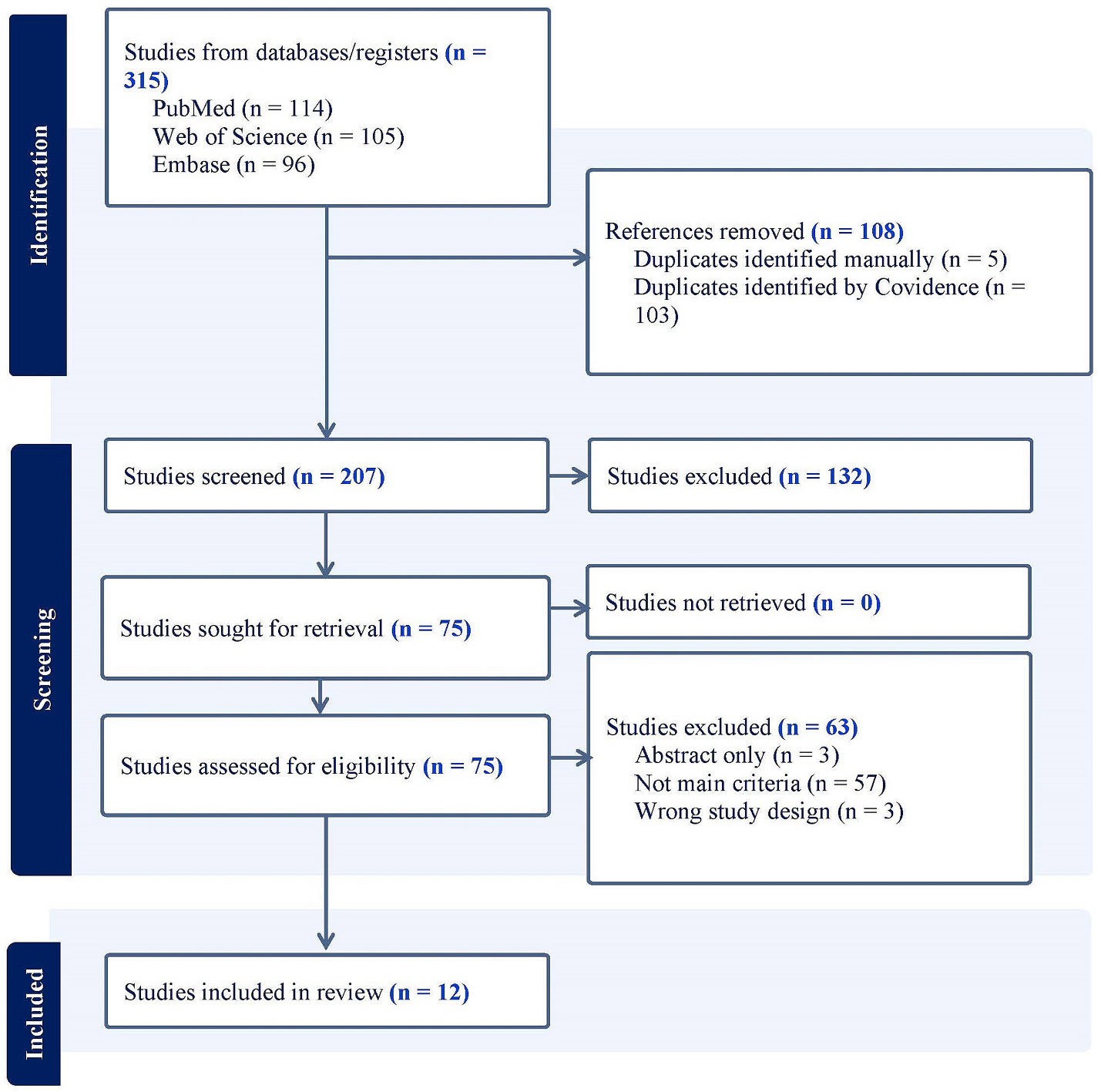
PRISMA diagram of literature search and selection process on systematic review on the Mendelian Randomization studies between Alzheimer’s disease or Parkinson’s disease and cancer. NOTE. This figure outlines the literature search strategy and selection process used in the systematic review of Mendelian randomization (MR) studies investigating the relationship between Alzheimer’s disease (AD), Parkinson’s disease (PD), and cancer.

The search and selection processes used in this systematic review are shown in the PRISMA Diagram (Fig. 1). Following a systematic literature search, we selected a total of 12 articles that met all inclusion criteria (Wu et al., 2021; Yuan et al., 2023; Dong et al., 2023; Huang et al., 2024; Lin et al., 2024; Li et al., 2024; Ren et al., 2024; Seddighi et al., 2020; Yu et al., 2024; Guo et al., 2023; Senkevich et al., 2021; Wang et al., 2023). Over 20 distinct types of cancers were assessed in the included MR studies, which examined the causal impact of cancers on AD or PD and/or the reciprocal impact of AD or PD on cancer risk. For AD-focused studies, bidirectional MR analyses investigated cancers, including colorectal cancer (CRC; also referred to as bowel cancer), endometrial cancers (with histological subtypes), follicular lymphoma, breast cancers, prostate cancer, oral cavity cancer, glioma, any neoplasms (benign or malignant), thyroid cancer, ovarian cancer (including subtypes), lung cancer, skin cancers, male-genital cancers, malignant neoplasms of the respiratory system and intrathoracic organs, and multiple myeloma (Table 1) (Wu et al., 2021; Yuan et al., 2023; Huang et al., 2024; Lin et al., 2024; Li et al., 2024).

In unidirectional MR analyses, the remaining cancers, such as renal cell carcinoma, pancreatic cancer, upper aerodigestive tract cancer, urinary bladder cancer, leukemia, lymphoma, generalized smoking-related and non-smoking-related cancers, and liver hepatocellular carcinoma (LIHC) were analyzed, treating them as either exposures or outcomes in relation to AD (Table 1) (Wu et al., 2021; Yuan et al., 2023; Huang et al., 2024; Lin et al., 2024; Li et al., 2024). Similarly, several cancers, including melanoma and ovarian, breast, prostate, endometrial, and keratinocyte cancers, have been investigated in bidirectional MR studies assessing their associations with PD (Table 2) (Lin et al., 2024; Guo et al., 2023; Senkevich et al., 2021). Chronic lymphocytic leukemia, CRC, cutaneous squamous cell carcinoma, lung cancer, lymphoma, glioma, oral and pharyngeal cancer, pancreatic cancer, and renal cell carcinoma were analyzed as exposures in a unidirectional MR study of PD. Although five studies (**Guo et al.**, **Lin et al.**, **Ren et al.**, **Senkevich et al.**, and **Wang et al.**) investigated the relationship between various types of cancers and PD (Lin et al., 2024; Ren et al., 2024; Guo et al., 2023; Senkevich et al., 2021; Wang et al., 2023), only one study (**Guo et al.**), which examined high-grade serous ovarian cancer (HGSOC) as an exposure and PD as an outcome, reported a significant inverse relationship (Guo et al., 2023).

The summary statistics utilized in these MR studies predominantly originate from populations of European ancestry, with data accessible through the Integrative Epidemiology Unit (IEU) GWAS database (https://gwas.mrcieu.ac.uk/). Sample sizes in each study ranged from 522 to 372,617 patients for cancers and from 15,056 to 218,792 patients for AD or PD. Detailed descriptions of the GWAS or studies are provided in their respective publications (Wu et al., 2021; Yuan et al., 2023; Dong et al., 2023; Huang et al., 2024; Lin et al., 2024; Li et al., 2024; Ren et al., 2024; Seddighi et al., 2020; Yu et al., 2024; Guo et al., 2023; Senkevich et al., 2021; Wang et al., 2023). Two studies by **Dong et al.** (Dong et al., 2023) and **Senkevich et al.** (Senkevich et al., 2021) reported results as regression coefficient estimates $\left(\hat{\beta }\right)$ and standard errors (SE), unlike other studies that reported OR and 95 % CI. To ensure consistency with the descriptions of other studies, $\hat{\beta }$ was converted to OR using the formula $\hat{OR}=exp(\hat{\beta })$ and 95 % of CIs were calculated using $CI=exp(\hat{\beta }\pm 1.96\times SE)$.

Details of the available GWAS studies, including sample sizes, total cases, and controls are summarized in Supplementary Table 2.

### 3.2. Significant findings by type of cancer for Alzheimer’s disease

Table 3 summarizes the significant associations between AD and various cancer types identified using the IVW method. Additional results, including non-significant findings from IVW and outcomes from other methods (e.g., MR-Egger, weighted median, simple mode, and weighted mode) are detailed in Supplementary Table 3. Notably, **Wu et al.** (Wu et al., 2021) reported only significant findings, applying a stringent significance threshold of *P* < 5 × 10^−4^ for robust associations, corresponding to a false discovery rate (FDR) < 0.05. Suggestive associations were reported for 5 × 10^−4^ < P < 5 × 10^−3^. **Wu et al.** (Wu et al., 2021) evaluated over 1000 CE-related risk factors, including more than 20 cancer-related GWAS, with only a subset meeting the stringent significance threshold. Non-significant and potentially spurious findings (e.g., those changing after the *APOE* region removal or with *P* > 0.05 in weighted median analysis) are presented in their supplementary materials and were excluded from our review to maintain a focus on robust, biologically meaningful associations. Descriptions of the significant findings by cancer type are provided in the sections below.

#### 3.2.1. Breast cancer

Consistent significant associations were observed in two MR studies (**Dong et al.** and **Ren et al.**) that examined breast cancer as an outcome, and in one study (**Seddighi et al.**) where breast cancer was evaluated as an exposure (Dong et al., 2023; Ren et al., 2024; Seddighi et al., 2020). Breast cancer GWAS summary statistics used in MR studies were obtained from the Breast Cancer Association Consortium (BCAC) and IEU (Medical Research Council Integrative Epidemiology Unit(MRC-IEU), 2025; Michailidou et al., 2017; The Breast Cancer Association Consortium (BCAC), 2025). Participants were screened according to national guidelines, and routine screening typically stopped after age 70–74 in cohorts such as the UK Biobank (Michailidou et al., 2017; NHS, 2025; Bycroft et al., 2018). Of the four MR analyses conducted by **Dong et al.** using four sets of GWAS summary statistics for breast cancer, only one MR analysis (GWAS ID: ukb-b-12,227), which included 10 genetic variants demonstrated that AD reduced the risk of breast cancer (OR = 0.997, 95 % CI = 0.994–0.999, *p* = 0.0043) based on the IVW method (Table 3).

Although the primary IVW analyses with the other three datasets yielded ORs < 1 that were not statistically significant, the weighted median method consistently produced significant results across all datasets (Supplementary Table 3). The results were further supported by no evidence of horizontal pleiotropy and heterogeneity, as indicated by the MR-Egger intercept and Cochran’s Q statistic (Supplementary Table 3) (Burgess and Thompson, 2017). In the reverse MR analysis, where breast cancer was set as the exposure and AD as the outcome, no significant causal relationship was found. However, the authors did not provide detailed results for this direction (i.e., cancer → AD), limiting our ability to interpret or include them in Supplementary Table 3.

**Ren et al.** found that AD significantly decreased the risk of estrogen receptor-positive (ER+) breast cancer and total breast cancer, based on the IVW analysis (Table 3) (Ren et al., 2024). However, after correcting for multiple comparisons, the protective effect of AD on overall breast cancer ceased to be significant (False Discovery Rate- (FDR) adjusted *p* = 0.099). In contrast, the protective effect of AD on ER+ breast cancer was further supported by various methods, such as MR-Egger, the weighted median, multivariable MR analyses, and FDR corrections (FDR-adjusted *p* = 0.032), with no evidence of directional pleiotropy or heterogeneity (Supplementary Table 3). There was no significant association between AD and ER-negative breast cancer (IVW: OR = 0.974, 95 % CI = 0.877–1.081, and *p* = 0.619). In a separate study **Seddighi et al.** using 109 genetic variants, reported that breast cancer patients were at low risk of developing AD (IVW: OR = 0.940, 95 % CI = 0.890–0.990, and *p* = 0.028), with MR-Egger intercept (*p*-value = 0.770) and Cochran’s Q (p-value = 0.153) that indicated no directional pleiotropy or heterogeneity (Seddighi et al., 2020).

However, these two significant MR findings should be interpreted with caution, as the breast cancer GWAS summary statistics were derived from the same consortium (BCAC) under different GWAS IDs and overlapping authors, which may reflect dataset dependency rather than independent replication (Supplementary Table 2).

#### 3.2.2. Colorectal cancer (CRC)

**Yuan et al.** (Yuan et al., 2023) conducted a bidirectional MR study to investigate the relation between CRC and AD, using two sets of AD summary statistics (discovery and validation). In forward MR analysis (i.e., the causal inference where CRC is hypothesized to influence AD), CRC was associated with a significantly reduced risk of AD, as indicated by the meta-analyzed results (Table 3). This protective association was consistently observed across both the discovery and validation samples and remained robust in sensitivity analyses (Supplementary Table 3). Additional methods, such as the MR-Egger, weighted median, and maximum likelihood (ML) approaches, yielded comparable results, further strengthening the evidence for a protective effect of CRC on AD (Supplementary Table 3). Conversely (i.e., AD → CRC), AD appears to increase the risk of developing CRC, as indicated by the meta-analyzed IVW results from two AD datasets (Table 3). However, these findings should be interpreted with caution due to potential sample overlap identified through the MRlap analysis and the absence of significant relationships in subgroup analyses within the AD validation cohort, as noted by the authors (Supplementary Table 3).

**Dong et al.** also utilized three distinct CRC GWAS summary statistics sets (GWAS IDs: ukb-b-17,001, ukb-a-296, and ukb-b-7748) to investigate the causal link between AD and CRC (Dong et al., 2023). In analysis using ukb-b-17,001, significant protective associations were observed with the IVW method (OR = 0.994, 95 % CI = 0.990–0.998, and *p* = 0.0036), weighted median (OR = 0.994, 95 % CI = 0.989–0.999, and *p* = 0.010), and weighted mode (OR = 0.994, 95 % CI = 0.989–0.999, and *p* = 0.0302). The MR-Egger (OR = 0.995, 95 % CI = 0.989–1.000, and *p* = 0.099) and simple mode (OR = 0.990, 95 % CI = 0.98–1.001, and *p* = 0.1336) methods did not show statistically significant results. Similarly, for ukb-a-296, the IVW method demonstrated a significant protective effect (OR = 0.993, 95 % CI = 0.988–0.998, and *p* = 0.0047), consistent with the weighted median (OR = 0.993, 95 % CI = 0.988–0.998, and *p* = 0.011) and weighted mode (OR = 0.993, 95 % CI = 0.988–0.998, and *p* = 0.032), although MR-Egger (OR = 0.994, 95 % CI = 0.988–1.001, and *p* = 0.112) and simple mode (OR = 0.992, 95 % CI = 0.982–1.003, and *p* = 0.207) results were not significant. In contrast, ukb-b-7748 showed significance, only in the MR-Egger (OR = 0.996, 95 % CI = 0.993–0.999, and *p* = 0.024) and weighted mode (OR = 0.997, 95 % CI = 0.995–1.000, and *p* = 0.049) methods, while the IVW (OR = 0.999, 95 % CI = 0.996–1.001, and *p* = 0.309) and other methods did not indicate a significant association. Overall, most odds ratios were less than one, suggesting a protective effect of AD on CRC, except for a non-significant positive association observed with the simple mode method in ukb-b-7748 (Supplementary Table 3).

#### 3.2.3. Endometrial cancer

**Dong et al.** (Dong et al., 2023) investigated the potential causal effect of AD on endometrial cancer, including both endometrioid and non-endometrioid histological subtypes. Having AD as an exposure significantly reduced the risk of endometrial cancer with endometrial histology using the IVW method (Table 3). This association was supported by other methods: weighted median (OR = 0.880, 95 % CI = 0.807–0.959, and *p* = 0.0037), MR-Egger (OR = 0.860, 95 % CI = 0.773–0.957, and *p* = 0.025), and weighted mode (OR = 0.878, 95 % CI = 0.807–0.956, and *p* = 0.015) (Supplementary Table 3). The simple mode method did not show statistical significance (OR = 0.878, 95 % CI = 0.712–1.083, and *p* = 0.256).

For non-endometrioid endometrial cancer, significant protective associations were observed with MR-Egger (OR = 0.704, 95 % CI = 0.533–0.930, *p* = 0.039) and weighted median (OR = 0.796, 95 % CI = 0.634–0.998, *p* = 0.048). However, the IVW method (OR = 0.844, 95 % CI = 0.670–1.063, *p* = 0.150), simple mode (OR = 0.938, 95 % CI = 0.530–1.660, *p* = 0.831), and weighted mode (OR = 0.779, 95 % CI = 0.622–0.976, *p* = 0.058) did not reach statistical significance (Supplementary Table 3). Overall, these findings suggest a potential protective effect of AD against both subtypes of endometrial cancer, particularly for the endometrioid subtype. In contrast, reverse MR analyses, treating AD as the outcome, found no significant associations across histological subtypes, with limited details reported in the original study and supplementary materials.

#### 3.2.4. Follicular lymphoma

The MR study by **Dong et al.** indicated a potential protective effect of AD on follicular lymphoma using 10 genetic instruments, although primary IVW was not significant (OR = 0.777, 95 % CI = 0.592–1.019, *p* = 0.068) (Dong et al., 2023). Both weighted median and weighted mode methods demonstrated a significant negative association: OR = 0.749, 95 % CI = 0.571–0.983, and *p* = 0.037 in the weighted median method and OR = 0.733, 95 % CI = 0.561–0.958, and *p* = 0.049 in the weighted mode method. In contrast, the MR-Egger (OR = 0.707, 95 % CI = 0.486–1.029, *p* = 0.107) and simple mode (OR = 0.556, 95 % CI = 0.275–1.125, *p* = 0.137) methods did not yield significant results (Supplementary Table 3). Tests for heterogeneity and horizontal pleiotropy indicated no violations of MR assumptions. These findings suggest a potential protective influence of AD against follicular lymphoma, although further validation is required. However, no significant results for the opposite direction (i.e., cancer → AD) were observed, and the author did not provide further information on the analysis.

#### 3.2.5. Prostate cancer

Four MR studies (**Dong et al.**, **Li et al.**, **Seddighi et al.**, and **Wu et al.**) were conducted assessing the relationship between AD and prostate cancer (Wu et al., 2021; Dong et al., 2023; Li et al., 2024; Seddighi et al., 2020). Among these, **Li et al.** reported a negative causal relationship when AD was analyzed as exposure and prostate cancer as the outcome (Table 3) (Li et al., 2024). Similarly, other methods also yielded significant results, including MR-Egger (OR = 0.974, 95 % CI = 0.953–0.996, and *p* = 0.039), the weighted median (OR = 0.975, 95 % CI = 0.957–0.993, and *p* = 0.007), and the weighted mode (OR = 0.975, 95 % CI = 0.957–0.993, and *p* = 0.020). In contrast, no significant causal relationship was found between prostate cancer and AD in reverse MR analysis (Prostate cancer → AD) (Supplementary Table 3). **Dong et al.** explored the causal relationship between AD as an exposure and prostate cancer as an outcome using two separate prostate cancer GWAS summary statistics sets: ieu-b-85 and ukb-b-7773. Although the IVW method did not yield significant results, the weighted median and weighted mode demonstrated significant negative associations (Supplementary Table 3). Conversely, when prostate cancer was treated as the exposure and AD as the outcome, conducted by **Dong et al.**, MR analysis showed no significant causal effect; and the results for MR (i.e., prostate cancer → AD) by **Dong et al.** were not provided in detail (Dong et al., 2023).

#### 3.2.6. Oral cavity cancer

Two bidirectional MR studies, conducted by **Dong et al.** and **Huang et al.**, investigated the causal relationship between AD and oral cavity cancer (Dong et al., 2023; Huang et al., 2024). **Dong et al.**’s primary IVW method in the forward MR (i.e., AD → oral cavity cancer) showed a significant decrease in risk (Table 3). The weighted median further confirmed this protective relationship (OR = 0.749, 95 % CI = 0.572–0.979, and *p* = 0.035). Other approaches, such as MR-Egger (OR = 0.732, 95 % CI = 0.530–1.011, and *p* = 0.100), simple mode (OR = 1.152, 95 % CI = 0.538–2.466, and *p* = 0.725), and weighted mode (OR = 0.744, 95 % CI = 0.556–0.994, and *p* = 0.080), did not yield significant results. In the reverse MR analysis (i.e., oral cavity cancer → AD), oral cavity cancer as an exposure was not causally related to AD as an outcome. Sensitivity tests and Cochran’s Q tests supported the robustness of these findings (Supplementary Table 3). **Huang et al.** reported that AD reduced the risk of oral cavity cancer. Using 34 genetic variants, they found a significant association via the IVW method with no evidence of horizontal pleiotropy and heterogeneity (Table 3 & Supplementary Table 3). The weighted mode (OR = 0.750, 95 % CI = 0.580–0.970, and *p* = 0.042) and weighted median (OR = 0.750, 95 % CI = 0.590–0.960, and *p* = 0.023) also consistently showed the protective effect of AD on oral cavity cancer. On the contrary, there was no significant association in the reverse direction MR analysis (oral cavity cancer → AD, *p* = 0.630).

#### 3.2.7. Glioma

**Wu et al.** investigated a comprehensive MR analysis using 1037 exposures and drugs for repurposing concerning AD as an outcome (Wu et al., 2021). The significance level was set to 5 × 10^−4^,which is equivalent to an FDR-adjusted *p*-value <0.05. Glioma had a significant positive relationship with AD (Table 3). However, they did not find a relationship between AD as an exposure and glioma as an outcome in the reverse direction MR analysis. Notably, the original article and supplementary materials did not provide results for the reverse direction.

#### 3.2.8. Neoplasms (benign or malignant - not specified)

**Wu et al.** (Wu et al., 2021) reported that neoplasms, as a grouped exposure, were associated with a reduced risk of AD, with the IVW method showing a significant effect, meeting the study’s suggestive significance threshold (Table 3). However, the extreme effect size and wide confidence interval suggest possible sparse data or weak instrumental bias. However, individual cancers (bronchus, lung, skin, prostate, respiratory, intrathoracic, breast (ER+, ER−), male genital organs, colon, rectum, anus, anal canal, ovary, bowel, melanoma, basal cell carcinoma, and low malignant potential) did not reach the significance threshold. Bidirectional MR was conducted in the study by **Wu et al.**; however, the results from the reverse MR analysis (i.e., AD → neoplasms) were not mentioned or provided in detail (Wu et al., 2021).

#### 3.2.9. Lung cancer

**Seddighi et al.** demonstrated that having lung cancer, one of the smoking-related cancers, showed a significant reduction in AD risk using 18 instrumental variables (Table 3) (Seddighi et al., 2020), suggesting a potential causal relationship with no significant heterogeneity (Cochran’s Q = 16.79 and *p* = 0.209) or pleiotropy (MR-Egger intercept = 0.024 and *p* = 0.118). Additional sensitivity analyses, including leave-one-out tests and funnel plots, confirmed the stability of the associations.

#### 3.2.10. Leukemia

**Seddighi et al.** also reported that leukemia was associated with reduced risk of AD in the conventional MR analysis using 38 instrumental variables (Table 3). Sensitivity analyses, such as the MR-Egger intercept test and Cochran’s Q test, indicated no pleiotropy or heterogeneity (Supplementary Table 3).

#### 3.2.11. Others

**Smoking-related cancers**: Smoking-related cancers, when analyzed collectively, were associated with a reduced risk of AD (Table 2). The MR-Egger intercept test (intercept = −0.003 and *p* = 0.695) confirmed the reliability of these associations, despite evidence of heterogeneity (Cochran’s Q = 84.73 and *p* = 0.006) (Supplementary Table 3), which may reflect variability in smoking effects across different cancer types (Seddighi et al., 2020).

**Non-smoking-related cancers: Seddighi et al.** reported that for cancers unrelated to smoking, each unit increase in the log odds of cancer was associated with a 1.9 % decrease in the odds of AD (Table 3). Sensitivity analyses, such as the MR-Egger intercept test, indicated no pleiotropy for non-smoking-related cancers. However, significant heterogeneity was observed (Cochran’s Q = 265.01 and *p* = 0.038) (Supplementary Table 3). Leave-one-out analyses were also used to validate the stability of the results (Seddighi et al., 2020).

**All (non-smoking and smoking-related) cancers:** A pooled analysis of genetic predictors for malignancies across all cancer sites revealed a 2.5 % decrease in risks of AD in the same study by **Seddighi et al** (Table 3). This finding suggests a consistent negative association between cancer and AD risk across a variety of cancer types, although significant heterogeneity was observed (Cochran’s Q = 353.53 and *p* = 0.002) (Seddighi et al., 2020).

**Liver hepatocellular carcinoma (LIHC):** In a multivariable MR (MVMR) analysis**, Yu et al.** showed a protective effect of AD against LIHC (Table 3), where AD was analyzed alongside platelet count, ambidextrousness, daily cigarette consumption, alcohol intake, and endocarditis (Yu et al., 2024).

### 3.3. Significant findings by type of cancer for Parkinson’s disease

#### 3.3.1. Parkinson’s disease and high-grade serous ovarian cancer (HGSOC)

A bidirectional MR study by **Guo et al.** explored the causal relation between ovarian cancers, including various histological types, and PD (Guo et al., 2023). The analysis revealed that HGSOC as an exposure was causally related to a decreased risk of genetically predicted PD, as shown by significant results using the IVW (OR = 0.910, 95 % CI = 0.840–0.990, and *p* = 0.030) and weighted median methods (OR = 0.890, 95 % CI = 0.810–0.990, and *p* = 0.020). However, no significant associations were found when PD was analyzed as the exposure and HGSOC as the outcome.

For overall ovarian cancer risk, no significant causal relationship was found with PD in either direction. The forward MR analysis yielded an OR of 0.800 (95 % CI = 0.610–1.060, *p* = 0.120), while the MR-Egger method showed an OR of 0.830 (95 % CI = 0.390–1.760 and *p* = 0.640). However, the weighted median method indicated a significant protective association (OR = 0.840, 95 % CI = 0.740–0.970, and p = 0.020). Across the remaining four MR studies, we found limited and inconsistent evidence of causal links between PD and several types of cancer (Supplementary Table 3) (Lin et al., 2024; Ren et al., 2024; Senkevich et al., 2021; Wang et al., 2023).

### 3.4. Assessment of core MR assumptions in included studies

To evaluate the methodological robustness of the MR studies included in our review, we assessed whether and how each study addressed the three core MR assumptions: “Relevance” (strength of association between the instrument and the exposure), “Independence” (absence of confounding between the instrument and the outcome), and “Exclusion Restriction” (no pleiotropic pathways between the instrument and the outcome). These assumptions are critical for valid causal inference using MR frameworks and serve as analogues to the assumptions underpinning randomized controlled trials (Davey Smith and Hemani, 2014).

A summary of the methods used by each included study to evaluate these assumptions is provided in Table 4. Most studies ensured instrument strength through SNP selection thresholds (e.g., *p* < 5 × 10^−8^) and F-statistics >10, and, when applicable, the inclusion of high-LD proxy SNPs (r^2^ > 0.8) to replace missing variants. However, some studies did not state whether proxies were used. “Independence” was generally addressed by restricting to European ancestry GWAS datasets, using linkage disequilibrium (LD) clumping, sensitivity analyses, and tools such as PhenoScanner (Kamat et al., 2019). “Exclusion Restriction” was typically evaluated using pleiotropy-robust MR methods, including MR-Egger intercept tests, MR-PRESSO, Cochran’s Q, leave-one-out analyses, and using Steiger filtering to exclude instruments that violate the directionality assumption.

While most studies addressed instrument strength through genome-wide significance and F-statistics, assessments of “Independence” and “Exclusion Restriction” were more variable. Some studies relied on theoretical justification, while others used pleiotropy-robust methods to partially address these assumptions.

Sample overlap primarily challenges the independence (no confounding) assumption of MR, as shared participants between exposure and outcome datasets can bias causal estimates. Among the included studies, Li et al. and Senkevich et al. explicitly addressed this issue—Li et al. provided detailed cohort descriptions, while Senkevich et al. used GWAS summary statistics that excluded potentially overlapping cohorts to minimize bias. Although cross-referencing GWAS sources (**Supplementary Table 4**) revealed no clear evidence of participant overlap, complete independence among large consortia cannot be fully assured; therefore, all results should be interpreted with caution.

Among the included MR studies, several instrumental SNPs were screened for associations with potential confounders such as smoking or alcohol consumption (Table 4), typically using PhenoScanner queries to identify and remove significant SNPs from both exposure and outcome datasets. Other studies addressed confounding indirectly through robust MR methods and sensitivity analyses. Only a few studies used MVMR, which allows genetic instrumental variables to be associated with multiple exposures and thus helps adjust for confounders (Sanderson, 2021). Although MVMR can provide more accurate estimates by accounting for the influence of several factors, its validity depends on the independence across all exposures. Consequently, the core MR assumptions remain uncertain in several studies, and their findings should be interpreted with caution.

## 4. Discussion

The review of 12 MR studies provides compelling evidence that AD is inversely associated with specific cancer subtypes, extending the findings from observational research. Notably, consistent inverse causal associations with AD were observed for colorectal, breast, endometrial, oral, lung, and prostate cancers, as well as leukemia, primarily supported by the IVW method. These results directionally align with previous observational studies reporting inverse relationships between cancer and neurodegenerative diseases. However, MR analyses strengthen causal inference by minimizing confounding and reverse causation, suggesting that these associations are unlikely to be entirely attributable to survival or detection bias(es). Conversely, MR studies did not replicate the broad protective associations for all cancer types observed in epidemiological cohorts, implying that part of the inverse relationship may arise from shared biological aging mechanisms, immune regulation differences, or selective survival rather than direct causality. Overall, while observational studies suggested a generalized protective effect of cancer against AD and PD (or vice versa), MR findings delineate cancer-specific, modest causal effects and emphasize that the inverse association with AD is not universal but specific to certain cancer types. In contrast, evidence supporting a causal relationship between PD and cancer was limited and inconsistent. Given the high prevalence of CRC/breast cancer and their shared relevance with AD in aging populations, our discussion focuses on these two cancer sites.

### 4.1. Biological mechanisms in neurodegenerative diseases and cancer

Neurodegenerative diseases and cancers have distinct underlying mechanisms. AD and PD are characterized by misfolded protein aggregates that trigger inflammation, neuronal stress, synapse elimination, and cell death (Moujalled et al., 2021; Radi et al., 2014). In contrast, cancers exhibit excessive cell proliferation and/or reduced apoptosis, along with aberrant DNA repair and, for solid tumors, angiogenesis (Lamptey et al., 2022; Adamu et al., 2024; Jan and Chaudhry, 2019; Lanni et al., 2021).

Despite these differences, when cancer and AD are considered overall, both are influenced by shared biological mechanisms with opposing outcomes. For example, apoptosis plays a paradoxical role in these diseases: excessive apoptosis has been suggested to contribute to neuronal death in AD, whereas resistance to apoptosis is a hallmark of at least some cancers, particularly chronic lymphocytic leukemia, demonstrating opposite pathological consequences (Jan and Chaudhry, 2019; Mattson, 2000; Billard, 2014; Loeder et al., 2009).

Additionally, abnormal cell cycle re-entry is another key factor in neuronal dysfunction and eventual cell death (Bonda et al., 2010). In healthy brains, neurons exit the cell cycle as they mature, enter the resting phase, and do not undergo further division. However, in AD-affected brains, neurons have been reported to attempt to re-enter the cell cycle in response to Aβ toxicity, tau-hyperphosphorylation, and inflammation (Norambuena et al., 2017; Barrett et al., 2021; Nandakumar et al., 2021). The aberrant cell cycle triggers DNA damage responses, ultimately leading to neuronal dysfunction, apoptosis, and neurodegeneration (Bonda et al., 2010; Barrio-Alonso et al., 2018). In cancer, dysregulated cell cycle re-entry drives uncontrolled proliferation and survival through multiple mechanisms. For example, cancer cells bypass quiescence, upregulate cyclin-dependent kinases, and overexpress cellular myelocytomatosis viral oncogene, facilitating sustained cell cycle progression (Zhang et al., 2019; Baker et al., 2022; Al-Qasem et al., 2022). Cancer cells also evade cell cycle checkpoints and apoptosis, enabling persistent division despite DNA damage (Suski et al., 2021). Notably, some cancers exhibit increased proliferation or apoptosis; however, they maintain their malignant phenotype because the rate of proliferation exceeds the rate of apoptosis.

**Fig. 2.**
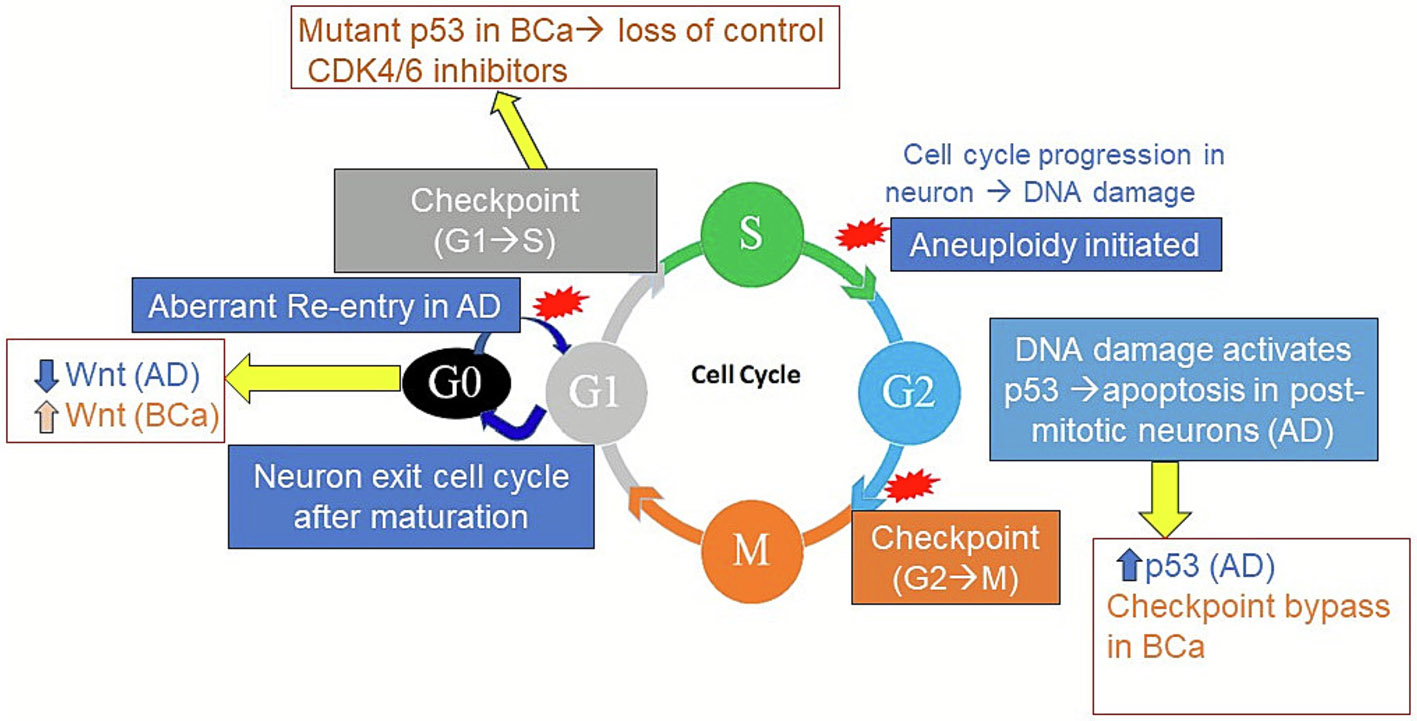
p53 and Wnt signaling at the crossroads of Alzheimer’s disease and breast cancer. NOTE. This schematic illustrates how p53 and Wnt signaling pathways interact to influence cell cycle progression in breast cancer (BCa) and Alzheimer’s disease (AD). G0, G1, S, G2, and M represent the cell cycle phases. Wnt activation promotes proliferation in BCa, while p53 signaling is implicated in neuronal apoptosis in AD. AD, Alzheimer’s disease; BCa, breast cancer.

The Wnt signaling pathway plays a crucial role in breast cancer, CRC, and, possibly, AD (Fig. 2) (Lanni et al., 2021; Tufail et al., 2025; Schatoff et al., 2017; Palomer et al., 2019; Manandhar et al., 2020; Caricasole et al., 2005). The Wnt activation is observed in over 50 % of breast cancer patients, correlating with poor overall survival (Tufail et al., 2025; Khramtsov et al., 2010; Xu et al., 2020) and promoting cell proliferation in CRC (Schatoff et al., 2017; Parker and Neufeld, 2020). Conversely, the Wnt inhibition is linked to heightened susceptibility to neurodegeneration (Palomer et al., 2019; García-Velázquez and Arias, 2017). Targeting Wnt signaling, such as GSK-3β inhibitors, shows promise in mitigating Aβ toxicity and neurodegeneration in preclinical AD models (Avila and Hernández, 2007; Griebel et al., 2019).

Hormonal signaling in breast cancer adds another layer of complexity to the relationship with AD. Both AD and breast cancer incidences increase with age and female sex, highlighting the role of estrogen decline in heightened AD risk (et al., 2024; Kulkoyluoglu-Cotul et al., 2019). Estrogen plays a crucial role in maintaining brain health through its effects on synaptic plasticity, mitochondrial function, and anti-inflammatory responses (Hara et al., 2015; Yao and Brinton, 2012; Mosconi et al., 2024; Vegeto et al., 2008). Emerging evidence positions that ER dysregulation serves as a key link between breast cancer and AD, with ERβ loss and ERα hyperactivation driving oncogenic and neurodegenerative processes, respectively (Bardin et al., 2004; Wang et al., 2016). These findings suggest that sex hormones may serve as a biological intersection between neurodegeneration and hormone-driven cancers.

The gut-brain axis has also emerged as a critical interface linking CRC and AD and gut microbiome dysbiosis implicated in AD through amyloid and lipopolysaccharides (LPS) secretion and blood-brain barrier disruption. However, the specific contribution of CRC-associated dysbiosis to AD remains largely unexplored. It may represent a key mechanistic link (Kesika et al., 2021; Kim et al., 2021; Escobar et al., 2022; Nguyen, 2022). One MR study reported a strong risk-reducing genetic correlation between cognitive and gastrointestinal (GIT) disorders, supporting the inverse relationship between cognitive traits and CRC (Adewuyi et al., 2022). However, factors like underdiagnosis of CRC in cognitively impaired patients, medication effects, and survival bias may influence the observed relationships. Conversely, some studies report increased risk of AD in CRC patients with vascular diseases or prolonged anesthesia (Akushevich et al., 2022; Du et al., 2022; Akushevich et al., 2021), highlighting the need for further research to clarify the underlying mechanisms and causality.

Additionally, the relationship between PD and cancers such as melanoma and glioma adds further nuance to the cancer-neurodegeneration axis. The MR study conducted by **Senkevich et al.** (Senkevich et al., 2021) found no causal relationship between PD and either melanoma or glioma, which contrasts with numerous epidemiological studies that report a positive association between PD and melanoma as well as gene expression overlap between PD and melanoma (Huang et al., 2015; Ryu et al., 2020; Dalvin et al., 2017). One possible explanation for the null MR findings is horizontal pleiotropy, where genetic variants influence PD and melanoma through independent, non-causal pathways. If these pleiotropic effects act in opposite directions, they could cancel each other out, resulting in no detectable causal signals. Similarly, no causal association was found between PD and glioma in **Senkevich et al.**’s MR analysis (Senkevich et al., 2021), despite some observational studies suggesting a positive relationship (Ye et al., 2016; Tang et al., 2016).

Several factors could explain this discrepancy. First, glioma is a relatively rare and highly heterogeneous tumor, which may limit statistical power in GWAS and weaken the strength of instrumental variables used in MR. Additionally, MR does not capture complex biological interactions, such as gene-environment interplay or epigenetic regulation, which may underlie associations observed in real-world settings.

### 4.2. Genetics in neurodegenerative diseases and Cancer

Several key genes including Peptidylprolyl Cis/Trans Isomerase, NIMA-Interacting 1 (*PIN1*) (Brokaw et al., 2020; Ajoolabady et al., 2022; Muth et al., 2019), tumor suppressors such as tumor protein p53 (*TP53*), retinoblastoma transcriptional corepressor 1 (*RB1*), and phosphatase and tensin homolog (*PTEN*) play pivotal roles in the biological mechanisms underlying both cancer and neurodegeneration processes (Farmer et al., 2025; Lanni et al., 2021; Abate et al., 2025; Nakanishi et al., 2025; Wolfrum et al., 2022; Chen et al., 2018; Fagiani et al., 2021; Indovina et al., 2015; Lee et al., 2018; Vidotto et al., 2020).

Loss-of-function mutations in *TP53* drive carcinogenesis by compromising its tumor-suppressive functions. These mutations cause cells to survive that should otherwise undergo apoptosis induced by DNA damage (Fig. 2) (Farmer et al., 2025; Nakanishi et al., 2025; Wolfrum et al., 2022; Tornesello, 2025). Conversely, excessive p53 activation disrupts cellular homeostasis by promoting senescence, altering metabolism, and influencing inflammatory responses (Farmer et al., 2025; Nakanishi et al., 2025; Wolfrum et al., 2022). As a key tumor suppressor, p53 is frequently dysfunctional in breast cancer and CRC, contributing to tumor progression (Fig. 1) (Farmer et al., 2025; Nakanishi et al., 2025; Wolfrum et al., 2022; Charni et al., 2017; Zilfou and Lowe, 2009). Beyond its canonical role in tumor suppression, dysregulation of p53 is also implicated in age-related pathologies and impaired tissue regeneration (Wolfrum et al., 2022; Charni et al., 2017; Zilfou and Lowe, 2009; Chen et al., 2022). Compelling evidence suggests that *PIN1* downregulation contributes to AD pathogenesis by driving tau pathology, synaptic dysfunction, and neurodegeneration. In contrast, *PIN1* upregulation promotes oncogenesis in multiple cancers, including breast cancer and CRC, highlighting opposing pathological outcomes (Chen et al., 2018; Fagiani et al., 2021). The loss of tumor suppressors such as *RB1* and *PTEN* promotes cancer development; however, their role in neurodegeneration is more complex, involving a delicate balance in neuronal health and function (Indovina et al., 2015; Lee et al., 2018; Vidotto et al., 2020).

Additionally, the amyloid precursor protein (*APP*)—the precursor of Aβ, a hallmark of AD—has been implicated in various malignancies, and *APP* overexpression has been reported in glioma (Greutter et al., 2024). These findings suggest a potential overlap in pathogenic pathways, which could offer common therapeutic targets. However, they also highlight the challenges in designing treatments that do not inadvertently increase the risk of one disease while targeting another.

Most MR studies have found no causal relationship between PD and cancers, except for one study reporting a weak inverse relationship with the histological subtype of ovarian cancer. A hallmark of PD is the progressive loss of dopaminergic neurons in the substantia nigra, which is responsible for dopamine production (Mhyre et al., 2012). This chronic and progressive neurodegenerative condition leads to a striatal dopamine deficit, impairing motor activities. PD is the second most common neurodegenerative disease after AD and is characterized by the presence of Lewy bodies, which are primarily composed of α-synuclein. The mechanisms underlying α-synuclein aggregation remain incompletely understood. Further research avenues are needed, especially given the rapidly growing prevalence of PD in recent decades and its substantial social and economic burden due to the long disease duration (Huang et al., 2015; Ryu et al., 2020; Dalvin et al., 2017; Ye et al., 2016; Tang et al., 2016).

A transcriptomic study comparing PD and site-specific cancers revealed that genes involved in mitochondrial processes, angiogenesis, and apoptotic activity are differentially regulated in these diseases (Forés-Martos et al., 2021). Furthermore, several PD-associated genes, such as *SNCA* (encoding α-synuclein, which forms the Lewy body aggregates found in PD), *PINK1* and *PARK7* (encoding PTEN-induced kinase 1 and DJ-1, respectively, both of which protect neurons from oxidative stress), and *PARKIN* (encoding a ubiquitin ligase), exhibit dual roles in cancer (Li et al., 2016). These genes can promote or suppress tumorigenesis through mechanisms involving mitochondrial dysfunction, oxidative stress, and protein aggregation, underscoring the complex interplay between PD and cancer (Ejma et al., 2020).

### 4.3. Risk factors for cancer and neurodegenerative diseases

Cancer and neurodegenerative diseases share multiple risk factors, including aging, lifestyle, environmental exposures, medical conditions, and social determinants (Plun-Favreau et al., 2010; Cannon and Greenamyre, 2011; Nabi and Tabassum, 2022). Aging is the most significant shared risk factor, with both conditions becoming increasingly prevalent in individuals aged 65 years or older (Plun-Favreau et al., 2010; Hou et al., 2019; Guo et al., 2022). Additionally, lifestyle factors such as smoking, alcohol consumption, poor diet, and physical inactivity, along with metabolic disorders, such as obesity and diabetes, promote chronic inflammation, oxidative stress, vascular dysfunction, and metabolic dysregulation, contributing to both diseases (Santiago and Potashkin, 2023). However, smoking presents a particularly complex relationship. Smoking increases the risk of lung and other cancers, while also being associated with vascular dementia and cognitive decline (Peters et al., 2008; Weber et al., 2021). In contrast, smoking reduces the risk of PD in some studies (Mappin-Kasirer et al., 2020; Cheng and Wang, 2020; Dorn, 1959). Chronic stress and hormonal imbalances (e.g., glucocorticoids, epinephrine) have been linked to Aβ plaque accumulation and autophagy disruption, exacerbating tumorigenesis and neurodegeneration (Santiago and Potashkin, 2023).

Despite these shared risk factors, several studies have reported an inverse relationship between cancer and neurodegenerative diseases (Driver et al., 2012; Musicco et al., 2013), suggesting distinct molecular mechanisms and offering potential therapeutic targets for both diseases.

### 4.4. Therapeutic effects

The impact of anti-cancer treatments on AD and the effects of AD treatments on cancer represent the final but crucial component of our discussion. Recent observational studies reported that chemotherapy may reduce the risk of AD in CRC patients for up to six years after diagnosis in a retrospective cohort study with 135,84 participants who are older than 65 years (Akushevich et al., 2021), raising the possibility of drug repurposing and shared molecular targets.

Specific chemotherapy agents, such as regorafenib, used in CRC treatment, have been shown to reduce neuroinflammation and amyloid-beta accumulation, potentially lowering AD risk in mouse models (Akushevich et al., 2021; Han et al., 2020). Interestingly, a large cohort study found that hormone-modulating therapy used to treat breast cancer was linked to a 7 % lower AD risk, with the most substantial effect in Black women aged 65–74 (24 %) vs. White women (11 %), highlighting age- and race-specific differences in its protective impact (Cai et al., 2024). Moreover, another study confirmed that breast cancer therapies lower the risk of AD by enhancing estrogenic pathways and activity in the brain through clinical, computational, and experimental analyses, demonstrating EMTs’ estrogenic effects on brain function, neuronal morphology, and mitochondrial bioenergetics (Branigan et al., 2023).

However, the timing and type of hormone therapy appear to be critical in determining its effects on AD risk. In the Cache County Study, which followed 1768 women over approximately 11 years, early-life hormone therapy use (within 5 years of menopause) is associated with a 30 % reduction in AD risk, while late-life initiation—particularly opposed to estrogen-progestin therapy—has been linked to increased dementia risk (Shao et al., 2012). Clinical trials such as the Women’s Health Initiative Memory Study (WHIMS) found that late-life opposed estrogen-progestin therapy increased dementia risk with hazard ratios (HRs) ranging from 1.93 to 2.05, reinforcing the critical window hypothesis, which posits that estrogen’s impact on AD risk depends on the timing and formulation of its use (Shao et al., 2012; Shumaker et al., 2004).

Additionally, Taxanes, including paclitaxel (Taxol), docetaxel (Taxotere), and cabazitaxel (Jevtana), are essential chemotherapeutic agents used in treating various cancers, such as breast, lung, and ovarian cancers, and advanced Kaposi’s sarcoma (Prota et al., 2023; Lindemann et al., 2012; Saville et al., 1995; Arbuck et al., 1994; Ettinger, 1993). These drugs stabilize microtubules, which has led to interest in their potential role in neurodegenerative diseases. However, most taxanes’ limited ability to cross the blood-brain barrier presents a significant challenge to repurpose for AD (Heimans et al., 1994).

A meta-analysis of breast cancer patients indicated some decline in domain-specific cognitive functions, and other observational studies reported that taxane-based chemotherapy could lead to “chemo-brain” (Ibrahim et al., 2021). Bexarotene, a retinoid X receptor (RXR) agonist, has been explored as a potential therapy for AD due to its role in Aβ clearance and neuroinflammation reduction. While preclinical studies demonstrated promising effects, clinical trials have yielded mixed and inconclusive results, primarily due to poor blood-brain barrier penetration, limited efficacy in *APOE* ε4 carriers, and safety concerns (Mezencev and Chernoff, 2020; Cummings et al., 2016; Leal et al., 2019; Ghosal et al., 2016). The details of the repurposed therapeutic effects of anti-cancer drugs, challenges, and clinical trial outcomes are extensively discussed in Ancidoni et al.’s systematic review (Ancidoni et al., 2021). Moreover, emerging evidence suggests that medications such as aspirin, metformin, and melatonin—widely used in cancer management—exert neuroprotective effects, including improved cognitive function and reduced chemotherapy-induced neuropathy (Mao-Ying et al., 2014; Laila et al., 2025; Palmer et al., 2020).

Further research priorities should include the integration of longitudinal cohort studies that combine systematic assessments for cancer diagnoses, treatment histories, and detailed cognitive phenotyping. Studies leveraging multi-modal brain imaging in cancer survivors—especially those exposed to chemotherapy or hormone therapies—could clarify neurobiological mechanisms underlying cancer–AD interactions. Additionally, large-scale biobanks and multi-ethnic datasets with genomic data are needed to explore gene–environment interactions and enable safer, personalized drug repurposing strategies.

### 4.5. Limitations in MR studies

Our discussion primarily focuses on the findings of IVW MR, which is considered the most powerful among MR methods when the assumptions are fulfilled and accounts for heterogeneity of variants. However, primary IVW is biased when horizontal pleiotropic effects are introduced. Many studies apply other methods, such as MR-Egger, the weighted median approach, and model-based techniques to identify and adjust for horizontal pleiotropy, which may distort the findings (Bowden et al., 2015; Hemani et al., 2018; Cho et al., 2020). The MR-Egger analysis relies on the Instrument Strength Independent of Direct Effect (InSIDE) assumption, which posits that the strength of genetic instruments (i.e., their associations with the exposure) is independent of any direct effects that they might have on the outcome (Burgess and Thompson, 2017). This InSIDE assumption is often difficult to verify fully and may not always hold, making some methods less reliable in certain situations (Lin et al., 2022). The weighted median- and mode-based estimations are also sensitive to outliers (Slob and Burgess, 2020). The weighted median method is imprecise when many instruments are valid, while the mode-based methods suffer from low precision power (Burgess et al., 2019). Although MR-PRESSO can remove outliers, it may produce misleading results when many instrumental variables are invalid. Recent studies have employed MR-RAPS, which is robust to horizontal pleiotropy and less sensitive to weak instruments (Burgess et al., 2019). Discrepancies between different MR approaches highlight the challenges of adequately addressing horizontal pleiotropy and invalid instruments. MR remains an emerging field, with new methods such as Contamination Mixture (Burgess et al., 2020) and MR-Mix (pleiotropy robust) (Qi and Chatterjee, 2019), MR-cML (Constrained Maximum Likelihood) (Xue et al., 2021), and CAUSE (Causal Analyses Using Summary Effect Estimates) (Morrison et al., 2020).

Many of these methods offer advantages over earlier techniques. Experts recommend specific MR methods according to step-by-step guidelines, especially when addressing uncorrelated and correlated pleiotropy (Slob and Burgess, 2020; Burgess et al., 2019; Sanderson et al., 2022). Despite the progress, each approach has strengths and limitations. When multiple MR methods produce consistent results within a study, the credibility of the causal inference is strengthened. However, discrepancies between methods do not necessarily invalidate causality; instead, they highlight the need for further investigation.

Furthermore, the use of GWAS summary statistics from populations of different races or ethnicities compared to target genetic data and the lack of sex-specific GWAS pose limitations to MR findings. Insufficient sample size reduces the statistical power necessary to detect causal relationships between diseases. Additionally, most MR studies do not explicitly account for survival bias or competing risks, which are particularly relevant for age-related diseases such as AD, PD, and cancer. These biases can affect genetic associations by selectively including individuals who have survived long enough to be studied, potentially distorting causal inference.

Another limitation in MR studies exploring the relationship between cancer, especially breast cancer, and AD is the risk of detection bias. Routine mammography screening typically ceases after age 70, and individuals with cognitive impairment are less likely to undergo cancer screening (NHS, 2025; Mehta et al., 2010). These factors increase the likelihood of undiagnosed breast cancer in older or cognitively impaired populations, which may contribute to the observed inverse associations between the two diseases. Therefore, MR findings should be interpreted with caution.

## 5. Conclusions and future directions

This review underscores that the relationship between AD/PD and cancer may be driven by cancer-specific mechanisms, particularly for breast cancers—where genetic findings align with observational data. This observation suggests that distinct biological pathways or interactions may underlie different neurodegenerative disorders and cancer types. A key limitation of current MR studies is that they do not account for shared biology. Methods like colocalization can help address this by determining whether the same genetic locus influences the exposure and outcome, especially since some SNPs may act as proxies through LD rather than exerting a direct effect. In addition to applying genome-wide significance thresholds, future MR studies investigating the relationships between AD/PD and cancers could adopt biologically informed strategies, such as selecting SNPs located within key cancer-related genes (e.g., *TP53, RB1, BRCA1/2, PIN1*) or relevant functional pathways. Using such targeted instruments may improve the biological interpretability of causal estimates and provide valuable insights into shared mechanisms linking cancer and neurodegeneration. As molecular profiling becomes routine in oncology, integrating known AD and neurodegenerative risk variants into clinical testing platforms could facilitate personalized cognitive risk stratification and survivorship planning. In the future, large GWAS that include diverse ancestry groups, sex-specific data, and integrated MR analyses—combined with colocalization and other functional approaches—will be crucial to improving causal inference. Ultimately, a deeper understanding of AD/PD and cancer relationships may ultimately inform survivorship care planning, guide cognitive screening strategies, and support personalized treatment decisions in elderly cancer patients at risk for neurodegeneration.

## Supplementary Material

1

## Figures and Tables

**Table 1:** Summary of Mendelian Randomization Studies on Alzheimer’s Disease and Cancer Relationships.

Cancer Type	Study								
	Dong et al., 2023	Huang et al., 2024	Li et al., 2024	Lin et al., 2024	Ren et al., 2024	Seddighi et al., 2019	Yu et al., 2024	Yuan et. al, 2024	Wu et al., 2021
Colorectal Cancer	E/O							E/O	
Endometrial cancer (endometroid/non-endometroid histology)	E/O								
Follicular lymphoma	E/O								
**Breast cancer**	**E/O**				**O**	**E**			**E/O**
**Breast cancer (ER+/−)**					**O**				**E/O**
Prostate cancer	E/O		E/O			E			E/O
Oral cavity cancer	E/O	E/O							
Glioma									E/O
Neoplasms									E/O
Thyroid cancer									E/O
ovarian cancers						E			E/O
Skin cancers									E/O
Lung cancer						E			E/O
Melanoma						E			E/O
Male genital cancers									E/O
Respiratory and intrathoracic organs cancer									E/O
Multiple myeloma				E/O					
Renal cell carcinoma						E			
Pancreatic cancer						E			
Upper aerodigestive tract cancer						E			
Urinary bladder cancer						E			
Smoking-related cancers (all)						E			
Leukemia						E			
Lymphoma						E			
Non-smoking-related cancers (all)						E			
Liver hepatocellular carcinoma							O		

Note: E, Exposure in MR analysis; O, Outcome in MR analysis; ER, estrogen receptor. Nine MR Studies on the relationships between AD and cancers were reviewed (Wu et al., 2021; Yuan et al., 2023; Dong et al., 2023; Huang et al., 2024; Lin et al., 2024; Li et al., 2024; Ren et al., 2024; Seddighi et al., 2020; Yu et al., 2024). Breast cancer was highlighted as it was the most consistently reported cancer type, showing an inverse association with AD.

**Table 2:** Summary of Mendelian Randomization Studies on Parkinson’s Disease and Cancer Relationships.

Cancer Type	Study				
	Guo et al., 2023	Lin et al., 2024	Ren et al., 2024	Senkevich et al., 2021	Wang et al., 2024
Colorectal cancer				E	
Endometrial cancer				E/O	
Lymphoma				E	
Breast cancer			O	E/O	
Breast cancer (ER+/−)			O		
Prostate cancer				E/O	O
Oral cavity and pharyngeal cancer				E	
Non-glioblastoma glioma/ glioma				E	
Ovarian cancer with different histological types	E/O				
Lung cancer				E	
Melanoma				E/O	
Chronic lymphocytic leukemia				E	
Multiple myeloma		E/O			
Pancreatic cancer				E	
Combined analysis of keratinocyte cancers				E	
Combined analysis of keratinocyte cancers				E	
Renal cell carcinoma				E	
Keratinocyte cancers				O	

Note: E, Exposure in MR analysis; O, Outcome in MR analysis; ER, estrogen receptor. Five MR Studies on the relationships between PD and cancers were reviewed (Lin et al., 2024; Ren et al., 2024; Guo et al., 2023; Senkevich et al., 2021; Wang et al., 2023).

**Table 3:** Significant Findings Between Cancers and Alzheimer’s Disease/Parkinson’s Disease Using Inverse Variant Weighting (IVW) Method.

Study	Exposure	Outcome	Number of SNPs	OR	95 % CI	P
	AD	Colorectal cancer	19	1.053	1.023–1.085	<0.001
	Colorectal cancer	AD	26	0.868	0.772–0.977	0.019
	AD (validation)	Colorectal cancer	47	1.006	0.991–1.020	0.443
Yuan et al.,2024	Colorectal cancer	AD (validation)	28	0.8	0.669–0.958	0.015
	AD	Colorectal cancer	(meta-analyzed)	1.014	1.001–1.027	
	Colorectal cancer	AD	(meta-analyzed)	0.85	0.762–0.929	
	AD	Endometrial cancer (endometroid histology)	10	0.906	0.832–0.984	0.014
Dong et al., 2023	AD	Breast Cancer *	10	0.997	0.995–0.998	0.0043
	AD	Oral cavity cancer *	9	0.766	0.601–0.976	0.0311
	AD	Colorectal cancer	10	0.994	0.990–0.998	0.0036
	AD	Colorectal cancer	10	0.993	0.9885–0.9979	0.0047
Huang et al., 2024	AD	Oral cavity cancer*	34	0.76	0.63–0.92	3.73 × 10^−3^
	Neoplasms	AD	10	0.060	0.01–0.30	6.3 × 10^−4^
Wu et al., 2021	Glioma	AD	3	1.130	1.06–1.21	4.8 × 10^−4^
Li et al., 2024	AD	Prostate cancer	13	0.974	0.958–0.991	0.003
Ren et al., 2024	AD	Breast Cancer*	86	0.925	0.871–0.982	0.011
	AD	ER+ breast cancer	86	0.912	0.853–0.975	0.007
	Lung cancer	AD	18	0.91	0.84–0.99	0.019
	Smoking-related cancers (all)	AD	68	0.95	0.92–0.98	0.003
Seddighi et al., 2019	Leukemia	AD	38	0.98	0.96–0.995	0.012
	Breast cancer	AD	109	0.94	0.89–0.99	0.028
	Non-smoking-related cancers (all)	AD	246	0.98	0.97–0.995	0.009
	All smoking and non-smoking-related cancers	AD	314	0.98	0.96–0.99	2.7 × 10^−4^
Yu et al., 2021	AD	Liver hepatocellular carcinoma (LIHC)	8(MVMR-IVW)	0.9999	0.9998–0.9999	0.01
Guo et al., 2023	High-Grade Serous Ovarian Cancer (HGSOC)	PD	11	0.91	0.84–0.99	0.03

NOTE. *Significant in two studies. SNP, single nucleotide polymorphism; OR, odds ratio; CI, confidence interval; ER, estrogen receptor; AD, Alzheimer’s Disease; PD, Parkinson’s Disease.

**Table 4:** Assessment of the three Instrumental Assumptions of Mendelian Randomization and the methods used for Validation.

Study	Relevance (F-statistic or IV strength, Proxy SNPs)	Independence (Confounding addressed)	Exclusion Restriction (Pleiotropy tested/ Heterogeneity/ MR-PRESSO/ Directionality confirmed)
Dong et al.,2023	SNP selection threshold (P < 5 × 10^−5^), LD clumping (done, details - not mentioned), F statistics >10, LD-Proxy: Minimum LD R-square value: 0.8.	European ancestry-only, addressed indirectly with multiple MR methods and heterogeneity tests (e.g., Cochran’s Q)	MR-Egger intercept test, Cochran’s Q, MR Steiger test
Huang et al., 2024	SNP selection threshold (*P* < 5 × 10^−8^), LD clumping (r^2^ < 0.001, clump window size of 10,000 kb), F statistics >10, Proxy SNPs (not mentioned)	European ancestry population, Relied on MR’s ability to reduce confounding because of random allocation of alleles at conception, indirectly addressed through sensitivity analysis and robust MR methods (IVW, weighted median/mode)	MR-Egger intercept test, MR-PRESSO, Funnel plot, and Cochran’s Q
Li et al., 2024	SNP selection threshold (*P* < 5 × 10^−8^), LD clumping (r^2^ < 0.001, clump window size 10,000 kb), F statistics >10, Proxy SNPs (LD: r2 > 0.8)	European ancestry-only, PhenoScanner, MR’s assumption (reduces confounding because of random allocation of alleles at conception), use of robust MR methods (median/mode), heterogeneity testing	MR-Egger intercept test, MR-PRESSO, Funnel plot, Cochran’s Q, MR Steiger test, Leave-one-out
Seddighi et al., 2019	SNP selection threshold (P < 5 × 10^−8^), Pairwise linkage analysis of SNPs and only independent SNPs (r^2^ < 0.2), Proxy SNPs (r^2^ > 0.9)	European ancestry-only, LD filtering (r2 > 0.8)	MR-Egger intercept test, funnel plots, leave-one-out sensitivity analysis
Yu et al., 2024	SNP selection threshold (P < 5 × 10^−8^), LD clumping (r^2^ < 0.001, clump window size 10,000 kb), F statistics, Proxy SNPs (not mentioned)	European ancestry-only, stringent LD clumping, LASSO regression, MR designs, robust MR methods	MR-Egger intercept test, MR-PRESSO, funnel plots, leave-one-out sensitivity analysis, Weighted median method.
Yuan et. al,2024	SNP selection threshold (P < 5 × 10^−6^), LD clumping (r^2^ < 0.001, clump window size 10,000 kb), F statistics >20, Proxy SNPs (LD: r2 > 0.8)	European ancestry only, PhenoScanner, Exclusion of SNPs linked to cofounders, Heterogeneity testing, MRlap (Sample overlap correction)	MR-Egger intercept test, MR-PRESSO, Leave-one-out analysis, Funnel plot, and Cochran’s Q, Simex Approach
Wu et al., 2021	SNP selection threshold (< 5 × 10^−8^), LD clumping (r^2^ < 0.001, clump window size 10,000 kb), F statistics (threshold not mentioned), Proxy SNPs (LD: r2 > 0.8))	European ancestry-only, Filtering for confounding by removing significant SNPs in both exposure and outcome datasets	MR-Egger intercept, MR-PRESSO, leave-one-out, weighted median method, APOE region exclusion
	SNP selection threshold (< 5 × 10^−8^), LD clumping (r^2^ < 0.001, clump window size 10,000 kb), Proxy SNPs (not mentioned)	European ancestry-only, MR designs	MR-Egger intercept, MR-PRESSO, Q-statistic, leave-one-out, weighted median method
Lin et al., 2024	SNP selection threshold (< 5 × 10^−8^), LD clumping (r^2^ < 0.001, clump window size 10,000 kb), F statistics >10, Proxy SNPs (not mentioned)	European ancestry only GWAS, PhenoScanner, harmonized and ambiguous palindromic SNPs excluded, excluded any exposure-correlated SNPs with p < 5 × 10^−8^ in the outcome	MR-Egger intercept test, MR-PRESSO, Cochran’s Q, Leave-one-out
Ren et al., 2024	SNP selection threshold (< 5 × 10^−8^), LD clumping (r^2^ < 0.1), F statistics >10	European ancestry only GWAS, PhenoScanner, harmonized, and ambiguous palindromic SNPs excluded	MR-Egger intercept test, Cochran’s Q, Leave-one-out, Multivariable MR
Senkevich et al., 2021	SNP selection threshold (< 5 × 10^−6^), LD clumping (r2 < 0.001, clump window size 10,000 kb), F statistics >10, Proxy SNPs (not mentioned)	European ancestry-only	MR-Egger intercept test, MR-PRESSO, Cochran’s Q, funnel plots, Steiger filtering
Wang et al., 2024	SNP selection threshold (< 5 × 10^−8^), LD clumping (r^2^ < 0.001, clump window size 10,000 kb), F statistics >10	European ancestry-only, PhenoScanner, Multivariable MR (MVMR)	MR-Egger intercept test, MR-PRESSO, funnel plots, Leave-one-out, Steiger filtering

NOTE. LD, linkage disequilibrium; SNP, single nucleotide polymorphism; IV, instrumental variable; MR, Mendelian randomization; IVW, inverse variance weighted; MR-PRESSO, Mendelian randomization pleiotropy residual sum and outlier; Q, Cochran’s Q heterogeneity test.

All studies used genome-wide significant or suggestive SNP selection thresholds (P < 5 × 10^−5^ or 5 × 10^−8^), LD clumping, and F-statistics >10 to ensure instrument strength. Confounding was addressed through ancestry restriction and robust MR methods. Pleiotropy was evaluated using sensitivity tests such as MR-Egger, MR-PRESSO, and funnel plots.

## Data Availability

All data used in this study are publicly available.

## Appendix A. Supplementary data

Supplementary data to this article can be found online at https://doi.org/10.1016/j.nbd.2025.107190.
